# How Does Dualistic Passion Fuel Academic Thriving? A Joint Moderated–Mediating Model

**DOI:** 10.3389/fpsyg.2021.666830

**Published:** 2021-06-07

**Authors:** Jie Zhou

**Affiliations:** Department of Police Management, Sichuan Police College, Luzhou, China

**Keywords:** dualistic passion, academic thriving, academic personal best goal, threat stress appraisal, academic workload

## Abstract

Based on the dualistic model of passion, this study developed a joint moderated–mediating model to investigate the mechanism of dualistic passion on academic thriving. We surveyed 960 Chinese university students with a questionnaire. The results showed that harmonious and obsessive passion positively predicted academic thriving, with the effect of harmonious passion being stronger. Academic personal best goal mediated these relationships. Moreover, threat stress appraisal and academic workload jointly moderated the direct effects of harmonious passion on academic personal best goal and obsessive passion on academic personal best goal, and the first stage of the mediating effects of academic personal best goal between harmonious passion and academic thriving as well as obsessive passion and academic thriving. Specifically, for low–threat stress appraisal and academic workload, the direct effect of harmonious passion on academic personal best goal and the mediating effect of academic personal best goal were stronger. Meanwhile, for high–threat stress appraisal and academic workload, the same applied for obsessive passion. These findings provide important implications for educational practice by highlighting an underlying mechanism of how and when dualistic passion, particularly for obsessive passion, can initiate and maintain academic thriving.

## Introduction

What motivates individuals to learn and work? Passion is a critical drive. Vallerand et al. (2003) defined passion as a strong inclination toward a specific activity that one loves, highly values, invests time and energy in, and is part of one's identity. They proposed the dualistic model of passion (DMP) and classified it into harmonious passion and obsessive passion in terms of how the activity in question has been internalized in the identity of an individual (Vallerand and Houlfort, 2003; Vallerand et al., 2003; Vallerand, 2015, 2016, 2020). The former refers to an autonomous internalization of the activity into one's identity and self, while the latter is a controlled internalization, and the level of control and pressure experienced when engaging in the activity is emphasized.

In the past two decades, the important influence of dualistic passion on individuals' adaptative and maladaptive outcomes has received extensive attention in positive organizational psychology (Liu et al., 2011; Zhang et al., 2014; Curran et al., 2015; Sirén et al., 2016; Jiang et al., 2017; Salessi et al., 2017). For example, studies revealed that harmonious passion is positively associated with adaptative workplace outcomes, including flow (Vallerand et al., 2003; Carpentier et al., 2012; Lavigne and Crevier-Braud, 2012; Thorgren et al., 2013), engagement (Ho et al., 2011; Birkeland and Buch, 2015), well-being (St-Louis et al., 2016), satisfaction (Thorgren et al., 2013), positive effect, performance (Dubreuil et al., 2014; Astakhova and Porter, 2015; Curran et al., 2015; Qadeer et al., 2016; Amarnani et al., 2020), work-family enrichment (Philippe et al., 2010), organizational citizenship behavior (Burke et al., 2015), and voice behavior (Gao and Jiang, 2019). Conversely, obsessive passion was more likely to lead to maladaptive workplace outcomes, including negative emotion (Stenseng et al., 2011), anxiety, depression, burnout (Vallerand et al., 2010a; Houlfort et al., 2018), emotional exhaustion (Fernet et al., 2014), work–family conflict (Vallerand et al., 2010a; Houlfort et al., 2018), and interpersonal distrust (Birkeland and Nerstad, 2015; Kong, 2016). Regarding research on dualistic passion in educational psychology, findings demonstrated that harmonious and obsessive passion influenced the outcomes of the students, including psychological well-being (Stoeber et al., 2011; Saville et al., 2018; Lin et al., 2019), physical health (Lafreniere et al., 2009; Forest et al., 2011; St-Louis et al., 2018), performance (Vallerand, 2016, 2020), creativity, and interpersonal relationships (Curran et al., 2015; Vallerand, 2016, 2020).

Nevertheless, among such numerous outcomes of dualistic passion, academic thriving in educational psychology is important but lacking attention. For university students, academic activities are an important part of university life, and they are devoting most of their time and energy in these activities. And some students engage in it out of love, while others do it because of external factors, such as exams, finding a good job, or winning a scholarship. These two types of passion in academic activities could lead to diverse academic outcomes, such as academic thriving (Stoeber et al., 2011; Saville et al., 2018; St-Louis et al., 2018). Based on the DMP (Vallerand and Houlfort, 2003; Vallerand et al., 2003; Vallerand, 2015, 2016, 2020) and socially embedded model (SEM) (Spreitzer et al., 2005), the resources derived from academic passion, such as positive affect, meaning, and motivation, can stimulate students' academic thriving. Hence, we suppose that passion in academic activities may have an important effect on academic thriving. However, previous researchers in the field of academic thriving mainly focused on its outcomes, less is known about its antecedents. In addition, researchers in the field of passion have paid much attention to its outcomes, whereas the relationship between students' dualistic passion in academic activities and academic thriving has been understudied.

In addition, if dualistic passion is closely related to academic thriving, then how this relationship occurs needs to be further explored. Based on the DMP, passion matters with teenage self-growth and development (Vallerand and Rapaport, 2017), such as vitality and flow (Vallerand and Houlfort, 2003; Ho et al., 2011), and this occurs by stimulating individuals' positive goal cognition (Zigarmi et al., 2009; Zhang et al., 2014). Academic personal best goal, a cognitive construct reflecting individuals' goals or standards of excellence that match or exceed one's previous best in the academic context, was significantly influenced by passion (Martin and Elliot, 2016a; Burns et al., 2018a,b) in academic activities and positively predicted engagement and achievement over time (Martin, 2011). For those harmoniously passionate students, they will set an academic personal best goal under strong autonomous motivation and positive emotion for self-growth; whereas for those obsessively passionate students, they will set an academic personal best goal under the psychological needs (Chénard-Poirier et al., 2021) of strong self-esteem and social acceptance for external rewards (Burns et al., 2018a). Thus, academic personal best goal may be a facilitator in the relationship between dualistic passion and academic thriving.

Finally, knowledge about the joint moderating effect of external and internal factors on the relationship between dualistic passion and academic thriving is limited. Is there any boundary condition that restricts this link? If these conditions exist, how do they work? According to the DMP (Moeller et al., 2017) and SEM (Spreitzer et al., 2005), the forming of academic thriving cannot be fully fueled with external or internal stimulation, and both play important roles in the forming process of academic thriving, such as external stressors and individuals' stress appraisals. Prem et al. (2017) found that challenge stressors, including time pressure and learning requirement, significantly predicted the sub-dimension of thriving—learning, and this effect worked through challenge and threat stress appraisals. Such that challenge stressors increased challenge stress appraisal, and in turn, fueled learning; and higher learning demands increased individuals' threat stress appraisal, and in turn, reduced vitality. In the present study, academic workload as a typical external stressor consisting of academic time pressure and learning demands, which is quite prevalent among university students' academic activities, and high workload may cause serious course stress, and then lead to threat stress appraisal (Vallerand, 2015, 2020; Smith, 2019). Moreover, such external stressors and internal threat cognition may have an interactive impact on academic thriving with dualistic passion through academic personal best goal. Thus, scholars appeal for taking academic stressor into consideration when investigating the effect of dualistic passion, as well as the interactive effect between academic stressor, stress appraisal style, and passion on academic outcomes (Bonneville-Roussy et al., 2010).

**Figure 1:**
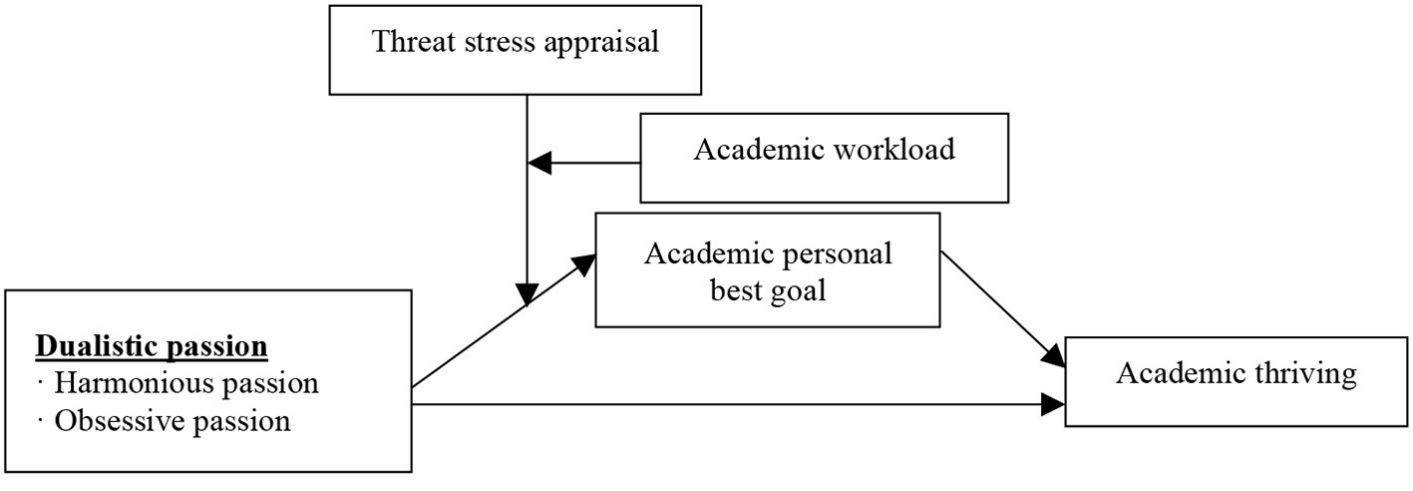
Theoretical model.

The purpose of this study was to explore the mechanism of the effect of dualistic passion on academic thriving. According to the DMP, we developed a joint moderated–mediating model to examine the direct effect of harmonious and obsessive passion on academic thriving, respectively, the mediating role of academic personal best goal in the above relationships, and the joint moderating effect of threat stress appraisal and academic workload on the direct effect of dualistic passion on academic personal best goal and the first stage of the mediating effects. By addressing these issues, this study may contribute to the theoretical framework of dualistic passion in education settings and to a better understanding of how and when dualistic passion influences academic thriving. Moreover, the present work has practical implications for educators to improve students' positive academic passion and outcomes. Our theoretical model is shown in Figure 1.

## Hypothesis Development

### Relationship Between Dualistic Passion and Academic Thriving

Academic thriving refers to a positive psychological state characterized by a joint experience of vitality and learning in academic activities; vitality represents positive emotions associated with energy and zest, and learning refers to the cognitive process of acquiring and applying new knowledge and skills in academic activities to improve ability and self-efficacy (Spreitzer et al., 2005; Porath et al., 2012). Thriving differs from engagement, flow, and flourishing, as it includes both energy and learning elements, while other concepts mainly focus on energy (Spreitzer et al., 2010; Zheng and Lu, 2013). Academic thriving highlights affect, meaning, knowledge, and other motivational resources of the individual's psychological experience in academic activities. The DMP considers that passion contains three elements, namely, affect (strong inclination), cognition (high value), and actual behavior (investment of time and energy) (Vallerand et al., 2003), which are consistent with the core content of thriving; thus, passion may stimulate students' academic thriving through these three elements.

First, dualistic passion provides positive affective resources for fueling academic thriving. Although harmonious passion and obsessive passion are autonomous and controlled drives, respectively, they both emphasize a strong affective inclination toward academic activity (Vallerand et al., 2010b; Vallerand, 2020). Dualistic passion plays a facilitator to heighten academic thriving by stimulating vitality (Porath et al., 2012) and engagement in academic activities (Kleine et al., 2019). Various studies have shown that harmonious passion is linked with positive emotion, which is positively related to thriving (Porath et al., 2012; Taneva and Arnold, 2018; Kleine et al., 2019), while obsessive passion is more likely associated with negative emotion, which encourages persistence in academic engagement (De Dreu et al., 2008), and such persistence can also fuel academic thriving (Schreiner, 2013).

Second, dualistic passion contributes to cognition resources (meaning) for improving academic thriving. According to the DMP, both harmonious and obsessive passion contribute to the meaning of academic activities, increasing the sense of meaning, importance, and concentration in relation to academic activities (Ho et al., 2018), expanding the scope of cognitive thinking and action; and enhancing cognitive ability to explore knowledge actively (Fredrickson, 2004); this, in turn, improves academic thriving (Siegel and Siegel, 2014). Stoeber et al. (2011) showed that dualistic passion was positively related to learning engagement, with harmoniously passionate students having higher dedication, obsessively passionate students having higher absorption, and both passions significantly enhancing vigor and reducing inefficacy. Research in the work context also found that a stronger sense of meaning and value at work was associated with higher energy and learning ability (Niessen et al., 2017).

Third, dualistic passion provides motivation resources for stimulating academic thriving. Thriving itself involves a strong inclination toward autonomous motivation, while harmonious passion, as an autonomous internalized passion, also involves a strong intention to act autonomously, which increases individuals' likelihood to invest time and energy in academic activities (Nix et al., 1999; Zigarmi et al., 2011; Dubreuil et al., 2014). In addition, obsessive passion, as a controlled internalized passion, will lead individuals to pursue high rankings, reputation, awards, and other external drives (Chénard-Poirier et al., 2021), and, in turn, will also increase time and energy investment (Yang et al., 2018). Therefore, the two types of passion involve behavioral motivation and will increase individuals' investment of time and energy in academic activities, thus igniting academic thriving. Accordingly, we proposed the following.

Hypotheses 1a−1b: Harmonious passion (1a) and obsessive passion (1b) positively predict academic thriving.

### Mediating Effect of Academic Personal Best Goal

Academic personal best goal refers to personalized goals or standards of excellence that match or exceed one's previous best in the academic context, and these goals are challenging, competitive, and specific (Martin and Liem, 2010). Based on the DMP, dualistic passion leads individuals to set academic personal best goals, as both harmonious passion and obsessive passion contribute to persistence and self-development in academic activities (Vallerand et al., 2010b). On the one hand, harmoniously passionate individuals will experience strong positive emotions and autonomous motivation regarding academic activities, being more likely to devote themselves to learning out of love (Zigarmi et al., 2009, 2011; Park et al., 2012), and achieving or exceeding more challenging goals for self-improvement and transcendence in academic activities (Martin and Elliot, 2016a; Burns et al., 2018a,b). When they reach or exceed their previous best goals, they will experience a strong sense of self-efficacy and self-satisfaction (Locke and Latham, 2002; Martin, 2011), and will then set another best goal. On the other hand, obsessively passionate individuals will set and achieve academic best goals to prove themselves to their parents, teachers, and peers, and obtain respect and more external rewards (Bélanger et al., 2013), which may further increase their engagement, even excessively (Vallerand and Houlfort, 2003). After satisfying the current needs of self-esteem and social acceptance, individuals driven by stronger external forces will set more best goals and strive to achieve or exceed them. In this way, an iterative process between dualistic passion and academic personal best goal develops (Burns et al., 2018a).

Furthermore, academic personal best goal may improve individuals' academic thriving. Individuals motivated by academic personal best goals mobilize available resources and focus their attention to achieve their goal, which is an important engine to stimulate focus (Cardon et al., 2009), flow experience, academic performance, and sustainable development (Martin, 2006). Setting academic personal best goals not only helps individuals to continuously invest energy in academic activities, increase classroom participation, and enhance learning motivation (Martin, 2011; Martin and Elliot, 2016a,b), but also provides specific information for individuals to achieve the “zone of proximal development” of best goals (Bandura, 1991). Reaching or exceeding their goals significantly enhances their sense of self-satisfaction and self-efficacy (Martin, 2013), stimulates higher goal commitment and achievement demands (Locke and Latham, 2002, 2013), promotes self-improvement, and enhances academic achievement and success (Burns et al., 2018a,b). Moreover, academic personal best goals are tailored to individuals' existing capabilities; thus, learning motivation and goal commitment will not decrease because the goal is too simple or difficult (Polivy and Herman, 2002; Locke and Latham, 2013). The challenge of achieving the best goals fitting individual ability is helpful to stimulate flow experience in academic activities (Csikszentmihalyi, 1990; Lavigne and Crevier-Braud, 2012). Setting and accomplishing the best goal is a positive and energetic iterative process that promotes continuous learning (Lin and Tsai, 2020) and mastering new knowledge (Zenasni and Lubart, 2011; Bledow et al., 2013; Hesselgreaves and Scholarios, 2014; Schoen, 2015; Burns et al., 2018a). Therefore, the vitality and learning experienced by individuals in this process also develop with the spiral rising circulation.

In addition, dualistic passion will improve academic thriving through academic personal best goal. According to the DMP, passion promotes motivation and positive goal cognition (Zigarmi et al., 2009; Zhang et al., 2014), and is accompanied by sustained and beneficial psychological well-being, including vitality and flow (Vallerand and Houlfort, 2003; Ho et al., 2011). High levels of harmonious passion and obsessive passion, respectively, reflect strong autonomous motivation and controlled motivation to set and achieve the best learning goals. Whether it is out of love for learning itself or driven by external factors, individuals are likely to set academic personal best goals under the influence of internal and external incentives, and increase their investment in the process of achieving or exceeding such goals; thus, they will experience more learning and vitality (Martin, 2007; Collie et al., 2016). In other words, academic personal best goal may mediate the translation of distal emotional motivation into proximal academic thriving. Accordingly, we proposed the following.

Hypotheses 2a−2b: Academic personal best goal mediates the respective relationship of harmonious passion (2a) and obsessive passion (2b) with academic thriving.

### Joint Moderating Effect of Threat Stress Appraisal and Academic Workload

Stress appraisal is a subjective evaluation process in which individuals conduct multiple and continuous evaluations of external stressors (Lazarus and Folkman, 1987), mainly regarding whether the stressor is challenging or threatening to their well-being. These appraisals determine different responses regarding emotion, motivation, and behavior, such that when external stressors are regarded as a challenge (challenging stress appraisal), individuals tend to adopt positive coping strategies, and when the stressors are perceived as a threat (threat stress appraisal), individuals are more likely to adopt emotion-oriented negative coping strategies (Skinner and Brewer, 2002).

In this study, we focused on threat stress appraisal and posited that it will affect the relationship between dualistic passion and academic personal best goal (Lepine et al., 2004). Specifically, individuals with high–threat stress appraisal regard difficulties and stressors in academic activities as threats, which can stimulate their obsessive passion controlled by external factors and weaken their internalized harmonious passion. Moreover, to avoid cognitive conflicts caused by cognitive dissonance, individuals are always striving for consistency between internal cognition and emotion, motivation, and behavior (Simon et al., 2004). The sense of tension, pressure, and negative emotion caused by high–threat stress appraisal is in line with the sense of control in high obsessive passion, which will strengthen the external driving force for individuals to set and achieve academic personal best goals, and will generate a positive spillover effect of obsessive passion on academic personal best goal. High–threat stress appraisal is contrary to the autonomic sense in high harmonious passion, and will thus reduce individuals' intrinsic drive to achieve and exceed previous best goals and will weaken the positive influence of harmonious passion on academic personal best goal.

In contrast, the cognitive dissonance between low–threat stress appraisal and harmonious passion is lower and will have lower interference with harmonious passion and the setting of academic personal best goals. Furthermore, the cognitive processing in obsessive passion is also lower in low–threat stress appraisal, and the inconsistency in cognition, emotion, and motivation between low–threat stress appraisal and high obsessive passion may inhibit the positive interaction effect of dualistic passion and threat stress appraisal on academic personal best goal. Hence, we proposed that threat stress appraisal moderates the relationship between dualistic passion and academic personal best goal.

Besides, the DMP also emphasizes the effect of external stimuli on individuals' stress evaluation and holds that emotion, motivation, and behavior are the results of internal factors, external environment, and the task itself (Moeller et al., 2017). Thus, we cannot examine the moderating effect of cognitive stress appraisal in isolation from specific situations (Wright et al., 2015). For most university students, academic workload, including course load, course difficulty, and time pressure, is an important and typical situational stressor that influences students' efficiency and academic status (Bonneville-Roussy et al., 2013; Northrup-Snyder et al., 2020; Vallerand, 2020). For instance, in a study with 1,299 university students in the UK, Smith's (2019) found that, compared with students with lower workload, those with higher workload and time pressure experienced higher learning efficiency and grade point average, but also greater course pressure and more negative psychological well-being, including life pressure, exhaustion, anxiety, and depression. Meanwhile, previous studies found that when individuals perceive greater pressure to complete a highly demanding task, they are more likely to strengthen their obsessive passion; on the contrary, when individuals perceive having resources (Barnett et al., 2012) for coping with work challenges, this is conducive to promoting their autonomous motivation internalization, which contributes to fueling harmonious passion Forest et al., 2012; Lavigne et al., 2014; Trepanier et al., 2014) and, in turn, may encourage individuals to set and exceed academic personal best goals. Hence, we inferred that academic workload, as an external situational stressor, will not only moderate the direct effect of dualistic passion on academic personal best goal, but will also further moderate the moderating effect of threat stress appraisal between dualistic passion and academic personal best goal.

More specifically, on the one hand, higher academic workload will strengthen the moderating effect of threat stress appraisal between harmonious passion and academic personal best goal, as well as weaken the moderating effect of threat stress appraisal between obsessive passion and academic personal best goal. When students perceive higher workload, the activation of threat stress appraisal is stronger. Under this condition, it is more likely for students' obsessive passion to increase because of the controlled drive (Mageau et al., 2009), and then, more anxiety, tension, and negative emotion will be induced (Matz and Wood, 2005; Jiang et al., 2019). Thus, individuals with higher obsessive passion will try to achieve or exceed their academic personal best goals to gain social acceptance, respect, and rewards. In contrast, when students' workload and threat stress appraisal are higher, the activation of harmonious passion caused by autonomic motivation is weaker. In this case, even if students have high harmonious passion, its positive influence on academic personal best goal is weaker. Therefore, despite stronger situational stimulus (higher academic workload) and higher threat stress appraisal, the strength of dualistic passion and its positive effect on academic personal best goal will still be lower, respectively.

On the other hand, low–academic workload will weaken the moderating effect of threat stress appraisal between obsessive passion and academic personal best goal, and will strengthen the moderating effect of threat stress appraisal between harmonious passion and academic personal best goal. When students perceive lower academic workload, the activation of threat stress appraisal is lower; in this case, the interference with harmonious passion is weaker. Thus, among the factors affecting academic personal best goals, the positive effect of harmonious passion is dominant, which is more conducive to setting and achieving academic personal best goals. In addition, when academic workload and threat stress appraisal are lower, although the dominant role of obsessive passion can promote setting academic personal best goals, its positive effect is weaker than when academic workload and threat stress appraisal are higher. Moreover, in the situation of higher academic workload, the effect of higher threat stress appraisal on obsessive passion and academic personal best goal is also weaker. Accordingly, we proposed the following.

Hypotheses 3a−3b: Academic workload and threat stress appraisal jointly moderate the relationship of harmonious passion (3a) and obsessive passion (3b) with academic personal best goal, respectively. Such that when academic workload and threat stress appraisal are low, the positive relationship between harmonious passion and academic personal best goal is stronger; while when academic workload and threat stress appraisal are high, the positive relationship between obsessive passion and academic personal best goal is stronger.

Finally, according to Hypotheses 2 and 3, we further proposed that academic workload and threat stress appraisal will jointly moderate the mediating effect of academic personal best goal between dualistic passion and academic thriving as follows.

Hypotheses 4a−4b: Academic workload and threat stress appraisal jointly moderate the first stage of the mediating effects of academic personal best goal between harmonious passion (4a) and obsessive passion (4b) and academic thriving, respectively. Such that when academic workload and threat stress appraisal are low, the mediating effect of academic personal best goal in the relationship between harmonious passion and academic thriving is stronger; while when academic workload and threat stress appraisal are high, the mediating effect of academic personal best goal between obsessive passion and academic personal best goal is stronger.

## Methods

### Participants and Procedure

In this study, we integrated field (paper questionnaire) and online surveys completed by university students from eight universities in western China. According to the previous studies (Lin et al., 2019; Chen et al., 2020; Jordan and Troth, 2020; Duffy et al., 2021), this study was conducted at three time points to minimize the common method bias (Podsakoff et al., 2003, 2012). First, we contacted principals of each university, and with their consent and support, students were invited to complete a paper questionnaire in a spacious and quiet classroom in each university at time 1. Students were informed that the research was anonymous and would not influence their evaluation of excellence, postgraduate recommendation, or graduation. After obtaining the students' consent, we administered the questionnaire. At time 1, there were 1,137 questionnaires distributed and collected, and demographic variables, dualistic passion, and academic workload were rated. At time 2 (2 weeks later), only academic personal best goal was rated, and 1,137 questionnaires were distributed online, and 1,100 questionnaires were returned. At time 3 (2 weeks later), only threat stress appraisal and academic thriving were rated, and 1,033 questionnaires were completed online.

After matching the questionnaires completed at the three time points, 960 valid responses were obtained after eliminating blank and invalid questionnaires with the same or missing answers, yielding an effective response rate of 84.83%. Of the 960 responders, 460 (47.92%) were male and 500 (52.08%) were female; 245 (25.52%) were majoring in science, 394 (41.04%) in engineering, and 321 (33.44%) in humanities and social sciences. Participants' distribution from freshman to senior grade was as follows: 95 (9.90%), 55 (5.73%), 195 (20.31%), and 615 (64.06%). A total of 120 (12.50%) respondents were from “985 Project” universities, and 840 (87.50%) were from regular universities. Generally, the “985 Project” universities are top and key supported universities by the Chinese government, whose quality of teachers and students is higher than that of regular universities, as well as the academic state and passion of students is better than that of regular ones. We chose students from the above two kinds of universities to highlight the diversity of samples.

### Measures

In this study, all measures were developed using established scales with good reliability and validity. All items were rated on a 5-point Likert scale ranging from 1 (*strongly disagree*) to 5 (*strongly agree*). The measures were as follows.

#### Dualistic Passion

The 14-item dualistic passion scale by Vallerand et al. (2003) was adopted in this study. It contains two dimensions: harmonious passion and obsessive passion, seven items for each dimension. For adaptation to university students in this study, we changed the expression “this activity” to “academic activities.” A sample item of harmonious passion was “Academic activities allow me to live a variety of experiences,” and a sample item of obsessive passion was “The urge is so strong. I can't help myself from engaging in academic activities.” The two original subscales in Vallerand et al. (2003) showed acceptable reliability (harmonious passion, Cronbach's α = 0.73; obsessive passion, Cronbach's α = 0.85) and validity with university students. In this study, Cronbach's α for harmonious passion and obsessive passion was 0.83 and 0.84, respectively.

#### Academic Thriving

We utilized the 10-item thriving scale by Porath et al. (2012), which consists of two dimensions: vitality (five items) and learning (five items), and it showed that the total reliability was 0.92 for the original scale. This scale has been widely used in occupational research and showed good reliability and validity in the Chinese context (Alikaj et al., 2020; Lin et al., 2020). In this study, we changed “At work” to “At academic activities,” to adopt items to the university context. A sample item for vitality was “At academic activities, I do not feel very energetic” (reverse coded), and a sample item for learning was “At academic activities, I find myself learning often.” In this study, Cronbach's α for the total scale was 0.84.

#### Academic Personal Best Goal

The 16-item academic personal best goal scale developed by Martin and Liem (2010) was used in this study, which consists of four dimensions, with four items in each dimension: specific goals (Cronbach's α = 0.90), challenging goals (Cronbach's α = 0.86), competitively goals (Cronbach's α = 0.88), and self-improvement goals (Cronbach's α = 0.82). Two sample items were “I set specific goals to aim for in my academic work,” and “I set challenging goals for myself in my academic work.” In this study, Cronbach's α ranged from 0.73 to 0.83 for the four dimensions and was 0.92 for the full scale.

#### Threat Stress Appraisal

We used the four-item threat stress appraisal scale developed by Drach-Zahvy and Erez (2002), which showed good reliability in the original scale (Cronbach's α = 0.89). A sample item was “Academic activities seem like a threat to me.” In this study, Cronbach's α was 0.80.

#### Academic Workload

The five-item academic workload scale developed by Wilson et al. (1997) (Cronbach's α = 0.74) was utilized in the present study. Two sample items were “The workload is too heavy” and “There's a lot of pressure on me as a student here.” In this study, Cronbach's α for this scale was 0.80.

#### Control Variables

In this study, we controlled for gender (male = 0, female = 1), university classification (“985 Project” university = 0, regular university = 1), major (science = major 1, engineering = major 2), and grade (grade 1 = 1, grade 2 = 2, grade 3 = 3, grade 4 = 4). Moreover, because of the self-report design and previous suggestions (St-Louis et al., 2020), we used four items of the social desirability scale developed by Reynolds (1982) to control for social desirability in this study; a sample item was “I have never intensely disliked anyone.” Cronbach's α for social desirability was 0.71.

### Data Analysis

The study used IBM SPSS 25.0 to perform descriptive statistics, correlation, and partial correlation analysis. Moreover, the model 11 in the PROCESS macro by Hayes (2013) was used to test hypotheses. In the model templates, model 11 assumed that the moderators Z (academic workload) and W (threat stress appraisal) jointly moderated the direct effect (harmonious passion and obsessive passion → academic personal best goal) and the first stage of the mediating effect (harmonious passion and obsessive passion → academic personal best goal → academic thriving), which was in accordance with our theoretical model. In addition, Amos 24.0 was used to perform the confirmatory factor analysis (CFA).

## Results

### Preliminary Analyses

#### Common Method Bias and Discriminant Validity

As previous studies detailed that self-report data and social desirability is a potential cause of artifactual variance in questionnaire research (Thomas and Kilmann, 1975; Podsakoff et al., 2003, 2012). The self-report design and key variables in this study may reflect some socially desirable attitudes, behaviors, and perceptions. Thus, according to the research design of this study and previous researches, we used Harman's single-factor analysis with CFA and the partial correlation analysis with social desirability as a measured markable variable (Podsakoff et al., 2003) to test the common method bias. The results of CFA showed that the single-factor model was a poor fit (χ^2^/df =9.04, RMSEA = 0.09, TLI = 0.50, CFI = 0.52). Moreover, the partial correlation analysis results demonstrated that compared with zero-order correlation coefficients, the first-order correlation coefficients only changed from 0.001 to 0.025, but the significance did not change significantly (Table 1). Thus, we acknowledged that common method bias existed in this study but was not serious. Besides, the research model (χ^2^/df = 2.96, RMSEA = 0.05, TLI = 0.88, CFI = 0.89) had a better fit than other alternative models, indicating an acceptable discriminant validity.

**Table 1:** Means, standard deviations, and correlation coefficients of variables.

**Variables**	**Zero-order correlation coefficients**								**First-order correlation coefficients**				
	***M***	***SD***	**1**	**2**	**3**	**4**	**5**	**6**	**1**	**2**	**3**	**4**	**5**
1. Harmonious passion	3.556	0.578	—						—				
2. Obsessive passion	3.146	0.640	0.588^**^	—					0.587^**^	—			
3. Academic thriving	3.566	0.515	0.467^**^	0.294^**^	—				0.465^**^	0.291^**^	—		
4. Academic personal best goal	3.507	0.514	0.474^**^	0.418^**^	0.503^**^	—			0.474^**^	0.417^**^	0.506^**^	—	
5. Threat stress appraisal	2.776	0.705	−0.167^**^	0.003	−0.328^**^	−0.149^**^	—		−0.159^**^	0.019	−0.296^**^	−0.149^**^	—
6. Academic workload	3.203	0.564	0.036	0.025	−0.013	0.121^**^	0.199^**^	—	0.045	0.033	0.012	0.127^**^	0.156^**^
7. Social desirability	3.115	0.667	−0.054	−0.043	−0.156^**^	−0.026	0.339^**^	0.159^**^					

* *p < 0.05*,

** *p < 0.01*.

*The first-order correlation coefficients mean correlation coefficients between variables after controlling for social desirability*.

#### Descriptive Statistics

The left part of Table 1 displays the means, standard deviations, and correlation coefficients for all variables. Harmonious passion was significantly and positively associated with obsessive passion (*r* = 0.588, *p* < 0.001), academic thriving (*r* = 0.467, *p* < 0.001), and academic personal best goal (*r* = 0.474, *p* < 0.001); and it was significantly and negatively associated with threat stress appraisal (*r* = −0.167, *p* < 0.001). Obsessive passion was significantly and positively associated with academic thriving (*r* = 0.294, *p* < 0.001) and academic personal best goal (*r* = 0.418, *p* < 0.001), and the coefficients between it and threat stress appraisal (*r* = 0.003, *p* > 0.05) were not significant. Academic thriving was significantly and positively correlated to academic personal best goal (*r* = 0.503, *p* < 0.001) and negatively correlated to threat stress appraisal (*r* = −0.328, *p* < 0.001). Academic personal best goal was significantly and negatively correlated to threat stress appraisal (*r* = −0.149, *p* < 0.001). Academic workload was significantly and positively correlated to threat stress appraisal (*r* = 0.199, *p* < 0.001) and academic personal best goal (*r* = 0.121, *p* < 0.001), but the coefficients between it and harmonious passion (*r* = 0.036, *p* > 0.05), obsessive passion (*r* = 0.025, *p* > 0.05), and academic thriving (*r* = −0.013, *p* > 0.05) were not significant.

### Hypothesis Testing

#### Direct Effects and Mediating Effects

All variables were standardized, and demographic variables as well as social desirability were controlled for in the equation. As shown in Table 2, in models 1 and 2, harmonious passion (β = 0.463, *p* < 0.001) and obsessive passion (β = 0.291, *p* < 0.001) positively predicted academic thriving, respectively, and the positive effect of harmonious passion was stronger, supporting Hypotheses 1a and 1b. In model 3, academic personal best goal positively predicted academic thriving (β = 0.496, *p* < 0.001), and when added it as a mediator, harmonious passion (β = 0.293, *p* < 0.001) and obsessive passion (β = 0.098, *p* < 0.001) still positively predicted academic thriving (see models 4 and 5), which demonstrated that academic personal best goal mediated the respective relationship of harmonious passion and obsessive passion with academic thriving, supporting Hypotheses 2a and 2b.

**Table 2:** Hierarchical linear regression results.

**Variable**	**Academic thriving**										**Academic personal best goal**			
	**Model 1**		**Model 2**		**Model 3**		**Model 4**		**Model 5**		**Model 6**		**Model 7**	
	**β**	**95% CI**	**β**	**95% CI**	**β**	**95% CI**	**β**	**95%CI**	**β**	**95%CI**	**β**	**95% CI**	**β**	**95% CI**
Gender	0.012	[−0.098, 0.144]	0.031	[−0.070, 0.193]	−0.018	[−0.154, 0.084]	−0.009	[−0.131, 0.095]	−0.008	[−0.135, 0.102]	0.059	[−0.004, 0.238]	0.071^*^	[0.016, 0.264]
Grade	0.002	[−0.061, 0.065]	−0.028	[−0.097, 0.039]	−0.018	[−0.079, 0.044]	−0.003	[−0.062, 0.055]	−0.020	[−0.081, 0.041]	0.039	[−0.024, 0.101]	0.016	[−0.048, 0.080]
University classification	0.100^**^	[0.117, 0.481]	0.091^**^	[0.075, 0.469]	0.071	[0.032, 0.388]	0.084^**^	[0.080, 0.420]	0.074^*^	[0.042, 0.397]	0.052	[−0.025, 0.336]	0.030	[−0.096, 0.272]
Major 1	0.097^**^	[0.068, 0.369]	0.114^**^	[0.095, 0.422]	0.095	[0.067, 0.362]	0.089^**^	[0.061, 0.342]	0.097^**^	[0.071, 0.365]	0.020	[−0.106, 0.194]	0.040	[−0.064, 0.242]
Major 2	0.077^*^	[0.011, 0.296]	0.090^*^	[0.025, 0.334]	0.064	[−0.012, 0.268]	0.063	[−0.007, 0.260]	0.066	[−0.007, 0.272]	0.028	[−0.086, 0.197]	0.029	[−0.086, 0.203]
Social desirability	−0.123^***^	[−0.178, −0.066]	−0.135^***^	[−0.194, −0.073]	−0.141^***^	[−0.194, −0.085]	−0.126^***^	[−0.177, −0.073]	−0.137^***^	[−0.190, −0.081]	0.021	[−0.037, 0.079]	0.039	[−0.022, 0.098]
Harmonious passion	0.463^***^	[0.407, 0.517]					0.293^***^	[0.234, 0.351]			0.441^***^	[0.381, 0.494]		
Obsessive passion			0.291^***^	[0.233, 0.354]					0.098^***^	[0.039, 0.159]			0.395^***^	[0.338, 0.454]
Academic personal best goal					0.496^***^	[0.444, 0.553]	0.357^***^	[0.300, 0.417]	0.455^***^	[0.397, 0.516]				
Treat stress appraisal											−0.077^*^	[−0.140, −0.015]	−0.174^***^	[−0.238, −0.113]
Academic workload											0.151^***^	[0.097, 0.213]	0.144^***^	[0.087, 0.208]
Harmonious passion × threat stress appraisal											−0.091^**^	[−0.144, −0.032]		
Harmonious passion × academic workload											0.003	[−0.048, 0.053]		
Threat stress appraisal × academic workload											−0.020	[−0.071, 0.035]	−0.016	[−0.066, 0.039]
Harmonious passion × threat stress appraisal × academic workload											0.134^***^	[0.053, 0.140]		
Obsessive passion × threat stress appraisal													−0.004	[−0.058, 0.051]
Obsessive passion × academic workload													0.011	[−0.043, 0.063]
Obsessive passion × threat stress appraisal × academic workload													0.130^***^	[0.049, 0.133]
*R*	0.502		0.350		0.533		0.591		0.540		0.525		0.491	
*R*^2^	0.252		0.123		0.284		0.349		0.292		0.276		0.241	
*F*	45.715^***^		19.026^***^		53.845^***^		63.845^***^		48.911^***^		27.689^***^		23.127^***^	

* *p < 0.05*,

** *p < 0.01*,

*** *p < 0.001*.

*Standardized regression coefficients were represented*.

#### Joint Moderating Effects

**Figure 2:**
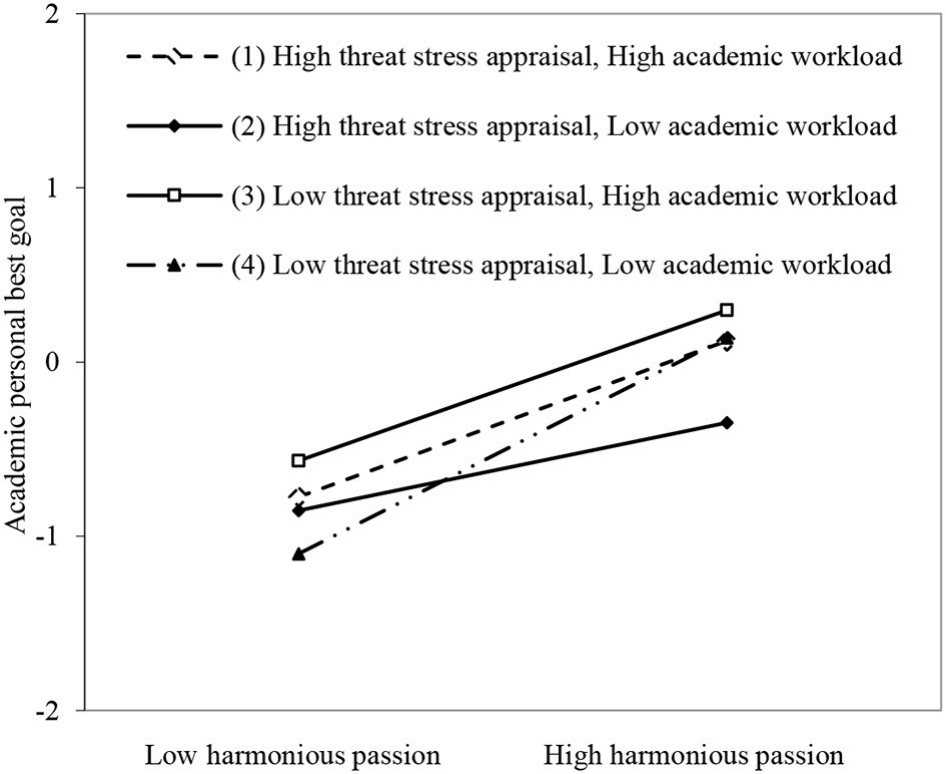
The joint moderating effect of threat stress appraisal and academic workload on harmonious passion and academic personal best goal.

As shown in model 6 in Table 2, the two-interactive effects of harmonious passion and threat stress appraisal on academic personal best goal were significant and negative (β = −0.091, *p* < 0.01). The two-interactive effects of academic workload and harmonious passion (β = 0.003, *p* > 0.05) and threat stress appraisal (β = −0.020, *p* > 0.05) were not significant. When controlling for the main effect and two-interactive terms, the three-interactive term of harmonious passion, threat stress appraisal, and academic workload positively predicted academic personal best goal (β = 0.134, *p* < 0.001). Simple slope results in Figure 2 show that when threat stress appraisal and academic workload were low (slope _low−low_ = 0.620, *p* < 0.001), the positive effect of harmonious passion on academic personal best goal was stronger than when threat stress appraisal and academic workload were high (slope_high−high_ = 0.448, *p* < 0.001), when threat stress appraisal was low and academic workload was high (slope_low−high_ = 0.432, *p* < 0.001), and when threat stress appraisal was high and academic workload was low (slope_high−low_ = 0.252, *p* < 0.001), supporting Hypothesis 3a. Furthermore, as shown in the left part of Table 3, the slope differences were significant between the group with high–threat stress appraisal and high academic workload vs. that with high–threat stress appraisal and low–academic workload (slope = 0.196, *p* < 0.01), and vs. that with low–threat stress appraisal and low–academic workload (slope = −0.172, *p* < 0.05). Moreover, the slope differences were significant between the group with high–threat stress appraisal and low–academic workload vs. that with low–threat stress appraisal and high–academic workload (slope = −0.180, *p* < 0.05), and vs. that with low–threat stress appraisal and low–academic workload (slope = −0.368, *p* < 0.001), and between the group with low–threat stress appraisal and high–academic workload vs. the group with low–threat stress appraisal and low–academic workload (slope = −0.188, *p* < 0.01).

**Table 3:** The significance test of slope difference.

**Model**	**Paired slope**	**Slope**	**95% CI**	**Model**	**Paired slope**	**Slope**	**95% CI**
Harmonious passion × threat stress appraisal × academic workload → academic personal best goal	(1) vs. (2)	0.196^**^	[0.064, 0.328]	Obsessive passion × threat stress appraisal × academic workload → academic personal best goal	(1) vs. (2)	0.202^**^	[0.072, 0.332]
	(1) vs. (3)	0.016	[−0.125, 0.157]		(1) vs. (3)	0.176^*^	[0.041, 0.311]
	(1) vs. (4)	−0.172^*^	[−0.304, −0.040]		(1) vs. (4)	0.014	[−0.118, 0.146]
	(2) vs. (3)	−0.180^*^	[−0.347, −0.013]		(2) vs. (3)	−0.026	[−0.194, 0.142]
	(2) vs. (4)	−0.368^***^	[−0.509, −0.227]		(2) vs. (4)	−0.188^**^	[−0.326, −0.050]
	(3) vs. (4)	−0.188^**^	[−0.322, −0.054]		(3) vs. (4)	−0.162^*^	[−0.301,−0.023]

* *p < 0.05*,

** *p < 0.01*,

*** *p < 0.001*.

**Figure 3:**
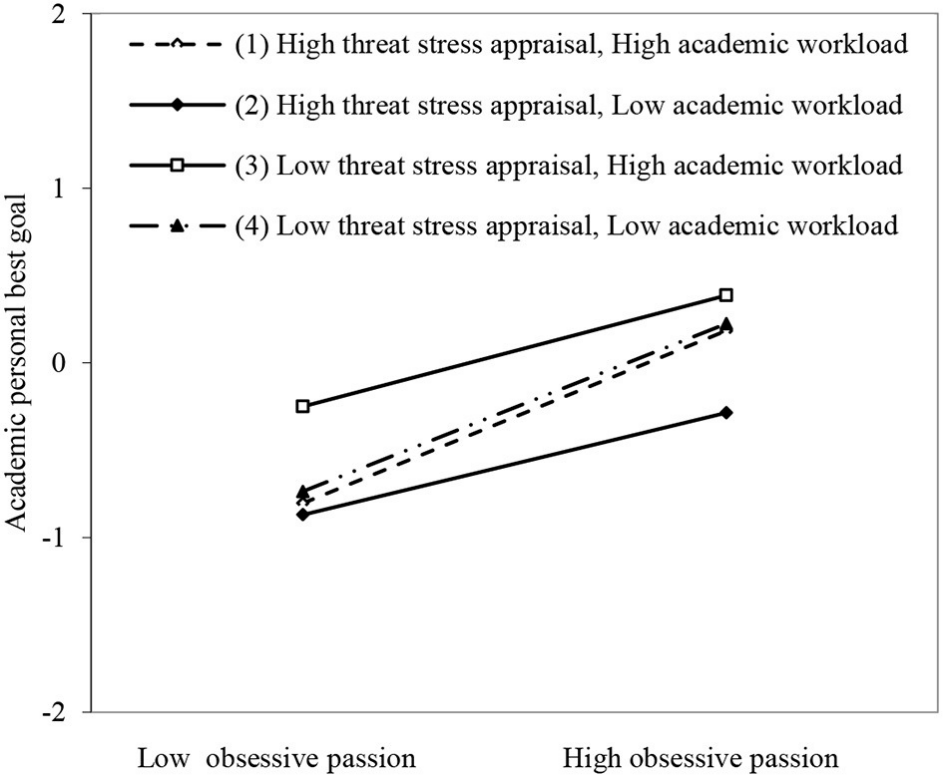
The joint moderating effect of threat stress appraisal and academic workload on obsessive passion and academic personal best goal.

Moreover, in model 7 in Table 2, all the two-interactive effects of obsessive passion and threat stress appraisal (β = −0.004, *p* > 0.05) and academic workload (β = 0.011, *p* > 0.05), as well as the two-interactive effects of threat stress appraisal and academic workload (β = −0.016, *p* > 0.05) on academic personal best goal were not significant. The three-interactive terms of obsessive passion, threat stress appraisal, and academic workload positively predicted academic personal best goal (β = 0.130, *p* < 0.001). As shown in Figure 3, when threat stress appraisal and academic workload were high (slope_high−high_ = 0.494, *p* < 0.001), the positive effect of obsessive passion on academic thriving was stronger than when threat stress appraisal and academic workload were low (slope_low−low_ = 0.480, *p* < 0.001), than when threat stress appraisal was low and academic workload was high (slope_low−high_ = 0.318, *p* < 0.001), and when threat stress appraisal was high and academic workload was low (slope_high−low_ = 0.292, *p* < 0.001), supporting Hypothesis 3b. Moreover, as shown in the right part of Table 3, the slope differences were significant between the group with high–threat stress appraisal and high–academic workload vs. that with high–threat stress appraisal and low–academic workload (slope = 0.202, *p* < 0.01), and vs. that with low–threat stress appraisal and high–academic workload (slope = 0.176, *p* < 0.05). Furthermore, the slope differences were significant between the group with low–threat stress appraisal and low–academic workload vs. that with high–threat stress appraisal and low–academic workload (slope = −0.188, *p* < 0.01), and vs. that with low–threat stress appraisal and high–academic workload (slope = −0.162, *p* < 0.05).

#### Joint Moderated Mediating Effects

Table 4 shows that the bias-corrected 95% confidence intervals (CIs) with 5000 bootstrap samples for moderated–mediating effects of harmonious passion → academic personal best goal → academic thriving (*B* = 0.035, 95% CI [0.015, 0.055]), and obsessive passion → academic personal best goal → academic thriving (*B* = 0.042, 95% CI [0.022, 0.061]) excluded zero. Besides, when threat stress appraisal and academic workload were low (*B* = 0.219, 95% CI [0.169, 0.271]), the mediating effect of harmonious passion → academic personal best goal → academic thriving was stronger than in other conditions; and when threat stress appraisal and academic workload were high (*B* = *0.2*23, 95% CI [0.169, 0.279]), the mediating effect of obsessive passion → academic personal best goal → academic thriving was stronger than in other conditions, supporting Hypotheses 4a and 4b.

**Table 4:** The joint moderated–mediating effect of academic workload and threat stress appraisal.

**Model**	**Moderator**		**Mediating effect**			**Moderated–mediating effect**		
	**Threat stress appraisal**	**Academic workload**	***B***	**SE**	**95% CI**	***B***	**SE**	**95% CI**
Harmonious passion → academic personal best goal → academic thriving	High	High	0.159	0.028	[0.107, 0.216]	0.035	0.010	[0.015, 0.055]
	High	Low	0.093	0.025	[0.044, 0.145]			
	Low	High	0.156	0.025	[0.108, 0.208]			
	Low	Low	0.219	0.026	[0.169, 0.271]			
Obsessive passion → academic personal best goal → academic thriving	High	High	0.223	0.028	[0.169, 0.279]	0.042	0.010	[0.022, 0.061]
	High	Low	0.136	0.029	[0.080, 0.193]			
	Low	High	0.149	0.025	[0.104, 0.202]			
	Low	Low	0.217	0.025	[0.169, 0.266]			

## Discussion

This study, drawing on the DMP, developed a joint moderated–mediating model to investigate how and when each type of passion in academic activities fuels university students' academic thriving. The results showed that both harmonious and obsessive passion positively predicted academic thriving, and the effect of the former was stronger. Moreover, academic personal best goal partially mediated these direct effects. In addition, threat stress appraisal and academic workload played a joint moderator role in the direct effects of harmonious and obsessive passion on academic personal best goal, as well as the first stage of the mediating effects.

### Theoretical Contributions

This study addresses an important but pretty much unexplored topic—linking dualistic passion with academic thriving, which makes three main contributions to the dualistic passion and thriving literature in the positive educational psychology field. First, it verified that DMP was a useful framework to explain the positive effect of harmonious and obsessive passion, particularly for obsessive passion, on academic thriving. In most previous studies, the mechanism of thriving followed the social embeddedness model (Spreitzer et al., 2010), and although the positive relationship and strength differences of dualistic passion with heavy energy and engagement among university students had drawn great attention (Stoeber et al., 2011). However, little was known about the effects of two types of passion, and whether their effects on academic thriving differ in strength under the theoretical framework of DMP. The present study addressed this research gap found that both harmonious and obsessive passion had positive impacts on academic thriving, with harmonious passion having a stronger effect. These findings expand the outcomes of dualistic passion and predictors of academic thriving, improve our understanding of the role of two types of passion in the process of fueling academic thriving. Moreover, the findings pave the way for researching other adaptive outcomes of dualistic passion underlying the framework of DMP in the educational field.

Second, the current research provides an insight into how dualistic passion translates into academic thriving by revealing the pivotal role of academic personal best goal. Research revealed that the two types of passion related to different goal orientations, and in turn, affected individual outcomes among psychological, sports, and music students (Vallerand, 2020). For example, mastery and performance goal orientations were found to mediate the relationship of each type of passion with performance (Verner-Filion et al., 2017) and interpersonal relationship (Guilbault et al., 2020). Although these goals involve self-growth, they are not about exceeding the best past performance, but mainly focus on doing better or avoiding doing worse than others, and there are few discussions on how dualistic passion affects academic thriving through academic personal best goal. Our study reveals the underlying mechanism of dualistic passion on academic thriving from the cognitive goal perspective by introducing academic personal best goal, which provides a new personal best goal setting-based perspective to understand how dualistic passion promotes academic thriving in the context of the Chinese university.

Third, this study increases our knowledge about when dualistic passion fuels academic thriving under the framework of the DMP by analyzing the joint moderated–mediating effect of threat stress appraisal and academic workload. Moeller et al. (2017) found that only ~20% of the variance in passion was explained by individual factors, the rest being accounted for by external factors. This work unpacks the “black box” of how and when multiple boundary conditions influence the mechanism of dualistic passion and academic personal best goal on academic thriving by revealing the joint moderated–mediating effects of threat stress appraisal and academic workload. The present study demonstrates that the sole focus on contextual factor and students' internal cognition may not be sufficient to account for the antecedents of academic thriving and shows that the forming process of academic thriving is more complex than it has been previously acknowledged. It also extends our understanding of the boundary conditions under which contextual (academic workload) and individual (threat stress appraisal) factors play a role in promoting academic thriving.

### Practical Implications

Our study also has important practical implications for students, teachers, and university organizations. First, given the positive effects of harmonious and obsessive passion on academic thriving in our findings, more interventions to identify students' strengths and improve their dualistic passion should be implemented. Previous research suggested that using one's strengths at work leads to an increased passion for work and more adaptative outcomes (Forest et al., 2012; Dubreuil et al., 2014, 2016); therefore, university students should first clarify their interests and strengths regarding academic activities (Peterson et al., 2005), as it is hard to find passion for academic activities without knowing oneself (Vallerand, 2020). Then, students could engage in academic activities after planting the seed of passion, particularly harmonious passion, toward a certain academic field. Moreover, teachers should also improve their passion and quality regarding teaching to fuel students' passion for academic activities (Ruiz-Alfonso and Leon, 2019). Besides, university organizations should create a positive teaching and learning environment for both teachers and students.

Second, our findings speak to educators who seek to better understand dualistic passion in a more comprehensive manner; in order to fuel students' academic thriving, educators should focus on the pivotal role of academic personal best goals in the relationship between dualistic passion and academic thriving, as this study found that academic personal best goals were conducive to fueling students' academic thriving and positively mediated the relationship between dualistic passion and academic thriving. Therefore, teachers should guide students to set personal best goals related to academic performance, and improve students' approach motivation; simultaneously, students should also autonomously set academic personal best goals that emphasize exceeding their previous best goals or standards rather than doing better or avoiding doing worse than others.

Finally, other boundary conditions related to students' academic performance such as stress appraisal style and workload should be taken into consideration according to individual students when educators want to help them set academic personal best goal and fuel their academic thriving by improving their dualistic passion. According to our findings, for harmoniously passionate students, a lower academic workload can reduce their threat stress appraisal, and such joint effect will subsequently strengthen the positive direct effect of harmonious passion on academic personal best goal and the indirect effect on academic thriving. In addition, for obsessively passionate students, a higher academic workload will increase their threat stress appraisal, further motivating their obsessive passion to set academic personal best goals, in turn fueling academic thriving.

### Limitations and Future Research

Despite the aforementioned contributions, this study also had some limitations that require further research. First, dualistic passion and thriving as a strong inclination and a psychological state, respectively, are dynamic rather than stable. Although we collected multi-time data, we could only investigate the static effect of dualistic passion on academic thriving, and causal relationships and their dynamic-mediating effect could not be established. Therefore, in the future, experience sampling and longitudinal designs (such as cross-lagged panel mediation model) should be encouraged. Second, environmental stressors (challenge and threat) will lead to two types of stress appraisal; however, this study only examined the effect of threat stress appraisal for one kind of threat stressor (i.e., academic workload), while the role of challenge appraisal style and stressors were not integrated into the whole framework nor controlled for. Future research should take them into consideration when exploring the relationship between dualistic passion and academic thriving *via* academic personal best goal. Third, teachers play an important role in students' academic performance (Vallerand, 2016, 2020), but this work did not explore the potential effect of teachers. Accordingly, it is necessary to investigate whether and how teachers' passion, support (Lalande et al., 2015), and teaching quality (Ruiz-Alfonso and Leon, 2019) may affect students' passion and other adaptative outcomes in academic activities. Finally, Astakhova (2015) found a significantly curvilinear relationship between harmonious passion at work and organizational citizenship behavior. Thus, whether harmonious passion and obsessive passion also have a curvilinear effect on adaptative outcomes in educational contexts needs to be further explored.

## Data Availability Statement

The datasets generated for this study are available on reasonable request to the corresponding author.

## Ethics Statement

Ethical review and approval were not required for the study on human participants in accordance with the local legislation and institutional requirements. The participants provided their written informed consent to participate in this study.

## Author Contributions

The author confirms being the sole contributor of this work and has approved it for publication.

## Conflict of Interest

The author declares that the research was conducted in the absence of any commercial or financial relationships that could be construed as a potential conflict of interest.
